# The Medical Profession and the Simplified Spelling1Read, by invitation, before the thirty-ninth annual meeting of the Medical Association of Central New York, held at Syracuse, N. Y., October 16, 1906.

**Published:** 1907-01

**Authors:** Burt G. Wilder

**Affiliations:** Ithaca, N. Y., Professor of Neurology and Vertebrate Zoology in Cornell University


### The Medical Profession and the Simplified Spelling.[1]

By BURT G. WILDER, M. D., Ithaca, N. Y.,
Professor of Neurology and Vertebrate Zoology in Cornell University.

"What attaches us to our conventional spelling is not a body of convictions, but simply habit and feeling." * * "Usage is another name for fashion, and fashions do not grow out of the ground or fall from Heaven, but are created by some one's initiative." Calvin Thomas.

"Language is an organism ; the organism that dislikes change is very ill, the one that fights change is moribund, and the one that does not change is dead." George M. Gould.

LIKE him who is challenged to a duel, he who is invited to address a society is accorded the privilege of choosing his subject. Yet, notwithstanding my keen personal interest in it (as akin to the Simplification of Anatomic Nomenclature that has occupied me largely for more than the fourth of a century)[2], I should refrain from exercising my prerogative in this particular direction but for my conviction that it is the duty of the members of our profession to recognize the existing defects of English spelling, to appreciate the past and present efforts to improve it, and to coöperate with the simplifiers in their individual, professional and associational capacities.

The defects of current English spelling fall under two heads, viz., (1) Superfluity of letters or of syllables,[3] involving waste of time, energy, space and money. The following examples, excepting the last, are selected from the "List of 300 Words" recommended in place of their longer forms by the Simplified Spelling Board and reprinted on a convenient card by the Govern-

1. Read, by invitation, before the thirty-ninth annual meeting of the Medical Association of Central New York, held at Syracuse, N. Y., October 16, 1906.

2. See, *e. g.*, "A partial revision of the nomenclature of the brain, Amer. Asso. Adv. Sci., 1880, *Medical Record*, vol. 18, p. 328. "Paronymy versus heteronymy as neuronymic principles," Address, as president, before the Amer. Neurol. Asso., 1885, *Transactions Jour. Nerv. and Mental Disease*, vol. 12 ; Neural terms, international and national," *Jour. Comp. Neurology*, vol. 6, 1896;" "Terminology, Anatomical" (with S. H. Gage), Reference Handbook of the Med. Sciences, 1st ed., vol. 8 ; "Some misapprehensions as to the simplified nomenclature of anatomy," Address, as president, before the Asso. Amer. Anatomists, 1898, *Science*, April 21st, 1899.

3. For this phrase a single word would be convenient. As suggested by a colleague, the Greek would supply perisso-grammatism, but that is lengthy and unfamiliar ; why not, after the analogy of *verbiage* and *verbosity*, (superfluity of words), *literage* or *literosity* ? The former is more acceptable in some respects, but the latter would warrant the adjective, *literose*, analogous with *verbose*.

ment Public Printer. The omitted superfluous letters are in italics. Catalog*ue*, dra*ch*m, *a*ether, hono*u*r, program*me*, synonym*e*, physiologic*al*. Certain cases will be considered later.

(2) Inconsistencies as to spelling and pronunciation that retard the educational progress of children and aliens and—upon the principle, *falsus in uno, falsus in omnibus*—tend to bring into contempt the language itself and the nations that use it. For example, *puff* is pronounced *puf;* but *rough* is pronounced *ruf,* yet *plough* is *plow, cough* is *korf, through* is *throo,* and *dough* is *do;* to cap the climax, *do* is pronounced *doo,* and *does* is either *duz* or *doz.* No wonder the alien is mystified and disgusted, and the child temporarily stultified; "there is engendered a disbelief in learning and a total lack of confidence in inference." Were it possible, indeed, for a rational adult to begin the study of his mother tongue he would speedily recognize the grim foundation for Lord Roseberry's recent half-humorous remark that he was "not at all sure that the archaic rules of spelling laid down by tradition and stereotyped by the dictionaries had not filled half the lunatic asylums of the country."

Referring to the Simplified Spelling Board's Circulars, 1-7, to the periodical, *Spelling,* and to various other publications for the fuller discussion of most points, upon the present occasion I confine myself to four groups of cases with which members of the medical profession are more nearly concerned and in respect to which their precept and example are likely to have most weight.

A. The conversion of Latin words ending in -ra and -rum into distinctively English paronyms in -er rather than in -re, which last is characteristically French; e. g., fiber, liter, and meter, like theater rather than theatre.[4]

B. The conversion of Latin (originally Greek) words ending in -mma into distinctively English paronyms by dropping the ultima; e. g., gram, diagram, program. The French "tail" is needless and incongruous.

C. In all Anglo-paronyms of Latin words containing ae or oe, the replacement of the diphthong by the single letter e; e. g., anemia for anaemia, esophagus for oesophagus.

In this connection it may properly be remarked that some who write foetus seem not to be aware that fetus also was used by the Romans; also, the would-be precisians who hold out for the diphthongs should make sure which is the right one in a given case; an extensive English work upon Zoology has coecum

---

4. For the definitions of *paronym*, *heteronym*, etc., see the papers named in Note 2 and "Some linguistic principles and questions involved in the simplification of the nomenclature of the brain," Amer. Philolog. Asso. Proceedings, vol. 36, pp. 14-19, 1905; copies of this paper will be sent upon application to the writer.

for caecum (better cecum) throughout, even in the index. Such pretentious pseudo-scholarship misleads others and excites derision in the better informed.[5]

D. With most Anglo-paronyms of Latin adjectives ending in -icus, the omission of the -al; e. g. anatomic and neurologic rather than anatomical and neurological.

Let me at once forestall adverse criticism by three concessions. (a) In a few cases, e. g., chemical, medical and surgical, the retention of the ultima does not render the entire word inconveniently long. (b) Altho correct in form *medic* now has a slangy sound and its admission to scholarly association may be delayed on that ground. (c) In official documents relating to associations whose original or corporate names contained the -al, it may be necessary to retain it, trusting (as suggested by me in a paper before the "American Philological Association" last winter) that the leading linguistic societies, here and abroad, may take the initiative in titular curtailment.

Meantime we are bound to regard the following conditions:—(1) With English adjectives the termination -al alone presupposes the Latin antecedent -alis, of which there are numerous examples. (2) The termination -ic alone presupposes the Latin antecedent -icus (Greek -ikos), and of this there are numerous examples. But the English termination -ical presupposes the Latin antecedent -icalis, and in classic Latin this does not occur so far as I am aware.

By whom and upon what grounds there were evolved the swarm of adjectives in -ical I know not. During the last half-century their number has materially diminished; we now say public, not publical, and we no longer follow Thackeray with heroical, St. John with epidemical, or Scott with aristocratical and enthusiastical. In the name of economy and sound etymology let us dispense with them all as rapidly as possible.

I regret that the Simplified Spelling Board did not deal sharply with this case. On the contrary, their ecumenical, while commendable in the replacement of the original dipthong by *e* constitutes an *apparent* sanction of all the -ical adjectives and practically counteracts the saving at the head of words like economical; doubtless, in time, this group of words will be duly considered.

Notwithstanding my dissent in the foregoing and a few other cases I bespeak for the recommendations of the Board the earnest consideration of the medical profession. The objections thus

5. The same error occurs in the text and index of the last edition of the leading German work upon Comparative Anatomy, altho absent from the English translations of two earlier editions.

far published seem to me either exaggerated or based upon an inadequate comprehension of what is proposed. Only one of them can be mentioned here. The Etymologic or Historic Objection, as has been well said, is often urged by those who are not themselves etymologists, and who could not, off-hand, give the origin and transformations of any given ten consecutive words. With the -ical adjectives it has been seen already that the etymologic evidence fails to justify the -al. But even were it otherwise, I hold that etymology and verbal history belong to special treatises and more comprehensive dictionaries, and that words, the tools of thought, should not be encumbered by etymologic superfluities; how many givers of alms are aware that the word is the scanty remnant of a Greek word, eleemosyna? The case is comparable to some in anatomy. Some still delight in pons Varolii and insula Reilii, altho there is no other pons and no other insula; had certain more familiar viscera been discovered or described by plain Englishmen, would that make it worth while to talk of the "stomach of Brown," the "intestine of Jones," or the "liver of Robinson?"

In general I feel assured that simplification of English spelling will avert waste in the present and for ourselves, and in still greater degree facilitate the educational progress of aliens and of coming generations.

In this busy age none has less time to waste than the medical practitioner. With no other class is altruism more nearly the dominating guide of life.

This remark, and previous ones as to the relations of the medical profession to Simplified Spelling apply equally to all English-speaking members. But it seems to me that the physicians of this country should be peculiarly mindful of the situation and of their opportunities and obligations. As American doctors they should be acquainted with and proud of the pioneer work in this direction by Dr. George M. Gould, editor of *American Medicine* and of standard medical dictionaries; since 1893 he has repeatedly and most forcibly expressed his convictions; his volume, "Suggestions to Medical Writers," 1900, is said to be now unobtainable; in my judgment no better use could be made of the sum required for its reproduction from the funds so wisely and generously provided by Mr. Andrew Carnegie. Secondly, as American scholars we should congratulate ourselves upon the existence of the Simplified Spelling Board, obtain its circulars (gratis) and study its recommendations. Thirdly, as American citizens, we should regard the executive order to the public printer to employ a certain set of 300 words. This action of the President of the United States lifts the whole matter from the serene depths of academic contemplation to the turbulent surface waters of general and vigorous discussion; to quote the words of President Roosevelt's sole living predecessor, now "it is not a theory that confronts us but a condition." To me, the president's order seems worthy to rank with his advocacy of the "strenuous life," with his declaration in favor of the "square deal," and with his achievement of peace between Russia and Japan.[6]

6. This estimate is increased rather than diminished by President Roosevelt's recognition of the right of the legislative branch to orthographic control of government documents. Trusting that a future Congress may take some steps toward the Simplified Spelling, let us hope that the present one may display an equally angelic "fear to tread" new paths in the event of a difference between this and some other power.

Dec. 15, 1906.

